# Uterine Rupture During Induced Abortion in the Second Trimester

**DOI:** 10.7759/cureus.76752

**Published:** 2025-01-01

**Authors:** Catarina Silva, Rita Palma, Rita Luz, Manuela Almeida, Antónia Santos

**Affiliations:** 1 Department of Obstetrics and Gynecology, Hospital Garcia de Orta, Almada, PRT

**Keywords:** medical termination of pregnancy, mifepristone, oral misoprostol, sulprostone, uterine rupture

## Abstract

Termination of pregnancy (TOP) in the second trimester accounts for less than 10% of all abortion procedures. Although it can be obtained by a surgical procedure, medical TOP is an effective and safe option. Prostaglandins have been used for cervical ripening in the second trimester but in the presence of uterine scars, the risk of uterine rupture rises. Given the increasing rate of cesarean deliveries worldwide, it is important to be aware of the risks associated with TOP in women with previous uterine scars. We describe a rare case, in a patient with a prior cesarean delivery, of a uterine rupture in a TOP at 20 weeks with a regimen of mifepristone-misoprostol and sulprostone.

## Introduction

Uterine rupture is an uncommon event during a termination of pregnancy (TOP). Even among women with a prior cesarean delivery, the risk of uterine rupture with misoprostol between 13 and 26 weeks is less than 0.3%. When mifepristone is given in addition to misoprostol for second-trimester TOP in women with a prior cesarean scar, the risk of uterine rupture rises to 1.15% [1-3]. Sulprostone (a prostaglandin E2 analog) or 16 phenoxy-omega-17,18,19,20 tetranor PGE2 methylsulfonylamide (a second-line uterotonic) is usually administered by intravenous infusion that induces a strong and sustained myometrial contraction [4]. These drugs can be found in some European countries like Portugal and there are some cases of uterine rupture reported [5,6]. This might be due to sulprostone being in disuse since misoprostol is an effective alternative to TOP. We present a case of uterine rupture at 20 weeks during a TOP with mifepristone, misoprostol, and subsequently sulprostone in a patient with a cesarean scar.

## Case presentation

A 33-year-old woman, Gravida 2 Para 1, was admitted for TOP due to multiple fetal anomalies at 20 weeks. She had a prior cesarean performed five years earlier at 38 weeks due to pre-eclampsia, with no other significant history of medical, gynecological, or surgical interventions.

The abortion was induced with oral mifepristone 200 mg, and 24 hours later, she initiated a regime of misoprostol and received two doses of misoprostol 400 mg (oral) at a five-hour interval. Afterward, the patient complained of abdominal pain, and she received an epidural. At this point, the cervix was ripe (4 cm of dilation and 70% effacement), and the induction progressed with amniotomy. A large quantity of hematic amniotic fluid was drained and a perfusion of sulprostone was initiated at a rate of 0.1 mg/hour (20 hours after the last dose of misoprostol). After 0.5 g of sulprostone, the cervix remained unchanged, and she complained about persistent pain in the right corner of the previous cesarean scar. On examination, she was conscious, normotensive (112/75 mmHg), with a normal heart rate (HR) of 80 bpm, and feverish (38 ºC). There was no vaginal bleeding. The abdomen was slightly tender at palpation on the right iliac fossa without abdominal guarding.

An emergency abdominal ultrasonography (US) revealed a dead fetus outside of the uterus contained by the broad ligament with retention of the fetal head inside the uterus. No free fluid was encountered in the pouch of Douglas or peri-hepatic and peri-splenic spaces.

Blood work at this point showed a hemoglobin (Hb) level of 12.8g/dL, with a slight elevation of white cells blood count (13.6x 106/L), neutrophils (83%), and C-reactive protein of 3.38 mg/dL. There were no other abnormalities in the complete blood count or coagulation test.

**Figure 1:**
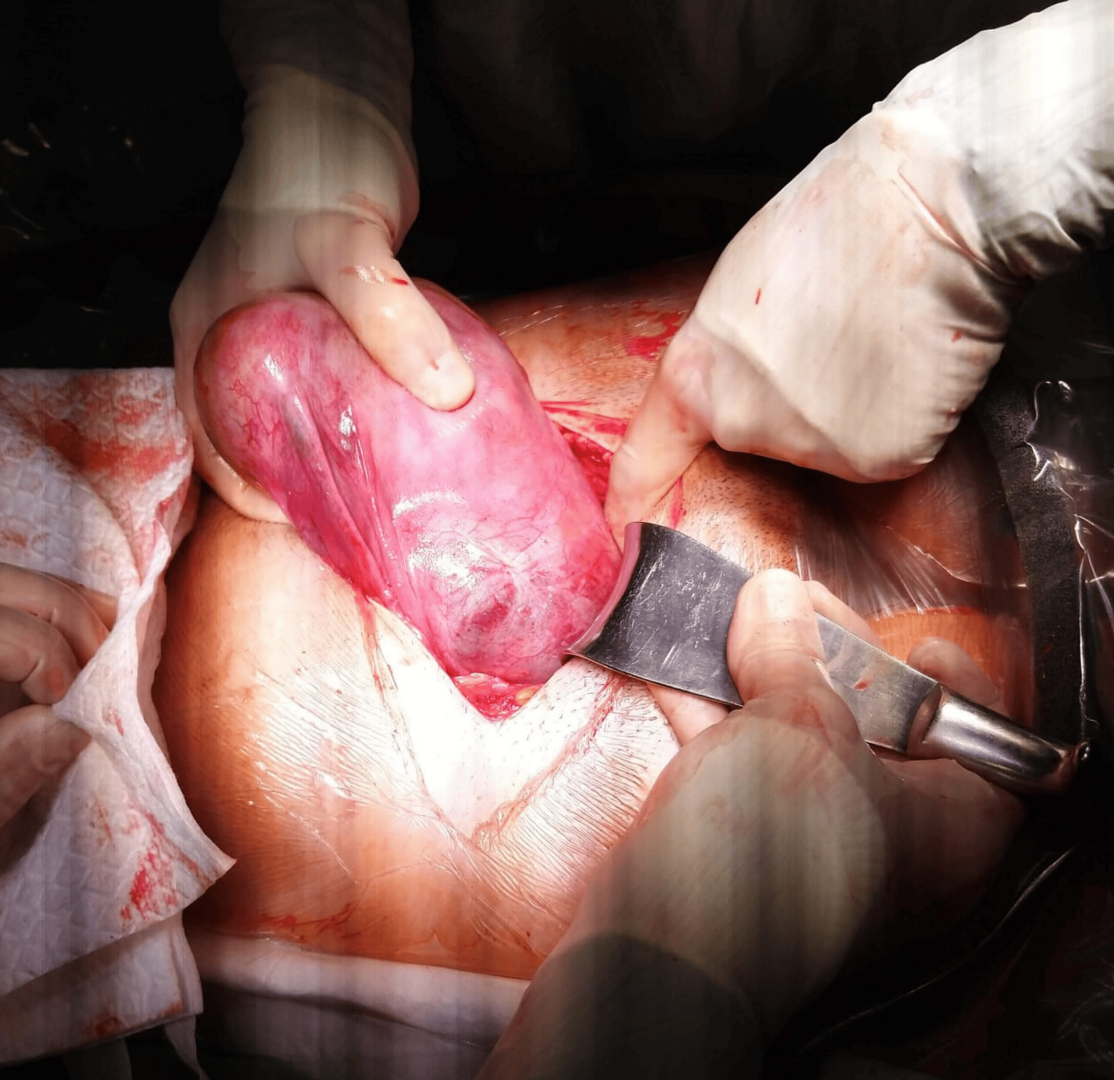
Bulging in the broad ligament due to the presence of fetal parts outside the uterine cavity

**Figure 2:**
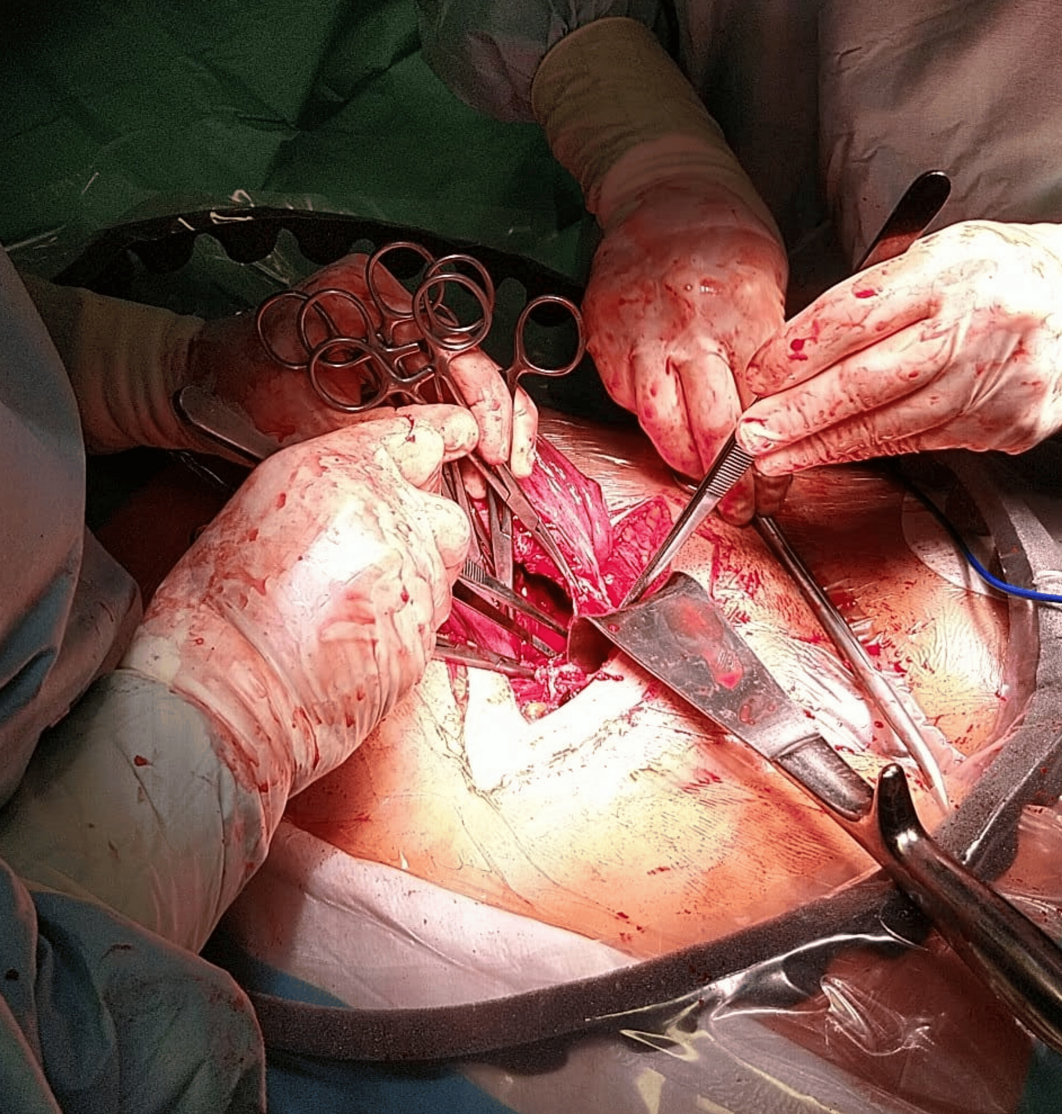
After opening the broad ligament, the uterine rupture was detected in the right corner of the prior cesarean scar (clamps on the edges and forceps showing the opening)

An emergency laparotomy was performed, assuming a uterine rupture. A right laceration of the uterine wall, approximately 6 cm in dimension, was identified during this procedure. The rupture had occurred in the right corner of the previous cesarean scar. The fetal body was contained by a broad ligament with no vascular or ureteric lesions associated. The broad ligament was opened, the fetal head was extracted, the placenta was removed by uterine massage and controlled cord traction, and the dehiscence was sutured. We used a continuous suture with a Vicryl suture 1.0. There was an estimated blood loss of 300 mL. The fetus weighed 274g (Figures 1, 2).

Recovery was favorable with no surgical complications. After this event, the patient became depressed, and psychological support was offered. We advised her to postpone a future pregnancy for 18 to 24 months to allow proper healing since our main concern was the recurrence of a uterine rupture.

Despite our recommendations, the patient became pregnant 12 months later. During pregnancy, she had a high risk of pre-eclampsia and was medicated with low-dose aspirin with poor compliance. At 29 weeks and 4 days, she was admitted due to a preterm rupture of membranes, and two days later, she had a cesarean delivery due to suspected fetal distress on cardiotocography. A male fetus was born at 29 weeks and 6 days in breech position, weighing 1200 g, with an Apgar score of 9 and 10 at the first and fifth minutes. There were no complications postpartum, and she was discharged three days later. The baby remained in the neonatal intensive care unit for 40 days due to his prematurity. With regard to neonatal morbidity, he developed hyaline membrane disease and maintained follow-up at the pediatric outpatient department.

## Discussion

During a TOP, women with uterine scars, such as from a cesarean delivery or myomectomy, have a higher risk of uterine rupture even though it is very uncommon [1-3]. In this case, we used mifepristone (a progesterone receptor antagonist) combined with a low dose of misoprostol (a prostaglandin E1 analog), followed by a low dose of sulprostone (a prostaglandin E2 analog) on the third day of TOP. Even though the mifepristone-misoprostol regimen is associated with an increased risk of uterine rupture, the overall risk is low [1], and this combination regimen is still the most effective for TOP between 14-0/7 and 27-6/7 weeks of gestation [7]. Therefore, in settings where mifepristone is available, misoprostol should be used in combination with mifepristone [8]. Some authors suggest that lowering the misoprostol dose in women with a prior CS after mifepristone priming may reduce uterine rupture [2].

Considering the half-life of misoprostol is approximately two hours [9], we concluded that the uterine rupture did not result from the combination of misoprostol and sulprostone (sulprostone was administered 20 hours after the last dose of misoprostol). One hypothesis is that the uterine rupture appeared after administering the combination regimen of mifepristone-misoprostol. The patient should have been reassessed to ensure that uterine rupture hadn't occurred when the epidural was offered, before changing the termination method to sulprostone. This highlights the importance of maintaining careful surveillance of women with a prior uterine scar under TOP. In the presence of intense persistent pain during TOP, the patient must be evaluated to rule out a uterine rupture, even in the absence of a hemorrhage [1,8,10].

Another aspect to attend to in these patients is their childbearing project. In 90% of cases, a hysterectomy can be avoided. If the uterus is preserved, the patients should be counseled about the risk of recurrence in future pregnancies. Although the overall risk of recurrent uterine rupture is 10%, if a patient with a history of uterine rupture intends to become pregnant, she must be aware of the risk and associated complications, including fetal and maternal death [11].

## Conclusions

The combination regimen of mifepristone and misoprostol is the most effective for TOP. Regardless of the increased risk of uterine rupture with this regimen, this event is still very uncommon. In this particular case, a regimen of mifepristone and misoprostol was used, which was shifted to sulprostone due to lack of progress. This highlights the importance of promptly reassessing patients with uterine scars in the event of sudden pain during TOP, even in the absence of vaginal hemorrhage, to exclude uterine rupture before changing methods of TOP or pain management. If uterine rupture is diagnosed quickly, conservative treatment of the uterus is possible in most cases, and the recurrence risk should be discussed with the patient.
